# Classic genetic and hormonal switches during fetal sex development and beyond

**DOI:** 10.1515/medgen-2023-2036

**Published:** 2023-08-16

**Authors:** Paul-Martin Holterhus, Alexandra Kulle, Hauke Busch, Malte Spielmann

**Affiliations:** Christian-Albrechts University of Kiel (CAU) Pediatric Endocrinology and Diabetes, Department of Pediatrics I Kiel Germany; Christian-Albrechts University of Kiel (CAU) Pediatric Endocrinology and Diabetes, Department of Pediatrics I Kiel Germany; University of Lübeck Medical Systems Biology Group, Lübeck Institute of Experimental Dermatology (LIED) Ratzeburger Allee 160 23562 Lübeck Germany; University of Lübeck Institute of Human Genetics Lübeck Germany

## Abstract

Critical genetic and hormonal switches characterize fetal sex development in humans. They are decisive for gonadal sex determination and subsequent differentiation of the genital and somatic sex phenotype. Only at the first glace these switches seem to behave like the dual 0 and 1 system in computer sciences and lead invariably to either typically male or female phenotypes. More recent data indicate that this model is insufficient. In addition, in case of distinct mutations, many of these switches may act variably, causing a functional continuum of alterations of gene functions and -dosages, enzymatic activities, sex hormone levels, and sex hormone sensitivity, giving rise to a broad clinical spectrum of biological differences of sex development (DSD) and potentially diversity of genital and somatic sex phenotypes. The gonadal anlage is initially a bipotential organ that can develop either into a testis or an ovary. *Sex-determining region Y (SRY)* is the most important upstream switch of gonadal sex determination inducing *SOX9* further downstream, leading to testicular Sertoli cell differentiation and the repression of ovarian pathways. If *SRY* is absent (virtually “switched off”), e. g., in 46,XX females, *RSPO1, WNT4, FOXL2*, and other factors repress the male pathway and promote ovarian development. Testosterone and its more potent derivative, dihydrotestosterone (DHT) as well as AMH, are the most important upstream hormonal switches in phenotypic sex differentiation. Masculinization of the genitalia, i. e., external genital midline fusion forming the scrotum, growth of the genital tubercle, and Wolffian duct development, occurs in response to testosterone synthesized by steroidogenic cells in the testis. Müllerian ducts will not develop into a uterus and fallopian tubes in males due to Anti-Müllerian-Hormone (AMH) produced by the Sertoli cells. The functionality of these two hormone-dependent switches is ensured by their corresponding receptors, the intracellular androgen receptor (AR) and the transmembrane AMH type II receptor. The absence of high testosterone and high AMH is crucial for anatomically female genital development during fetal life. Recent technological advances, including single-cell and spatial transcriptomics, will likely shed more light on the nature of these molecular switches.

## Genetic switches of fetal sex development

Sex determination of the gonads is typically associated with 46,XX sex chromosomes in females leading to ovaries and 46,XY sex chromosomes in males leading to testes. Initially, the gonadal anlagen are pluripotent in both sexes. Transcription factors like *WT1* transcription factor (also known as Wilms-tumor 1) [1–3], *Chromobox 2* (*CBX2)* [4],as well as *Nuclear receptor subfamily 5 group A member1* (*NR5A1,* also known as *SF1*) [5] are expressed at these early stages in the indifferent gonad. The relevance of these genes for early gonadal development is well documented by their association with distinct biological differences of sex development (DSD) due to deletions and mutations leading to gonadal dysgenesis [1–5]. At the end of the 6^th^ week of gestation, *Sex determining Region Y (SRY)* is the most important upstream “master switch” of gonadal sex determination. If *SRY* is expressed at certain high threshold levels, testicular differentiation is “switched on,” and ovarian differentiation is repressed [6, 7]. Interestingly, transgenic expression of *Sry* in XX-mice induces testis development [8]. Accordingly, in the human *SRY* translocation to the X-chromosome induces XX-maleness and inactivating mutations of *SRY* lead to 46,XY gonadal dysgenesis [9, 10], also called Swyer syndrome. The most important downstream target of SRY is *SRY-box 9 (SOX9)*, an *SRY-*related HMG box factor [11] that is expressed in the developing Sertoli cells [12]. Mutations or deletions of *SOX9* leads to DSD due to gonadal dysgenesis. Moreover, they are combined with campomelic dysplasia due to expression of *SOX9* in the developing cartilage [13, 14]. Interestingly, *SOX9* overexpression can lead to 46,XX testicular DSD independent of the presence of *SRY* [15, 16]. Regular testicular determination involves further genes like *NR5A1*, *desert hedgehog (DHH),* and mastermind like domain containing 1 (*MAMLD1)* supporting Leydig cell differentiation and proper steroidogenesis [17–20].

The absence of *SRY* expression and, therefore also the subsequent absence of upregulation of *SOX9* in 46,XX individuals allows to switch on ovarian development. An important mechanism promoting ovarian determination is *Wingless-Type MMTV integration site family (WNT)* signaling antagonising *SOX9*, preventing testicular development. Therefore, *R-spondin 1 (RSPO1), WNT4, and ß-catenin (CTNNB1)* act together as an important switch to establish regular ovarian development [21, 22]. In XX mice, knockout of either *Rspo1* or *Wnt4* leads to a similar phenotype with partial sex reversal, and ectopically activated testosterone biosynthesis [21, 23]**,** and in humans, mutations in either *RSPO1* or *WNT4* have been associated with 46,XX DSD underlining the importance of these genes for ovarian development. *Forkhead box L2 (FOXL2)* is involved in regular granulosa cell development and its lifelong maintenance. Inducible deletion of *Foxl2* in adult ovarian follicles of mice, i. e. if it is virtually “switched off”, leads to transdifferentiation of the ovary into the testes [24]. On the other hand, loss of *double sex and mab-related transcription factor 1 (dmrt1)* in adult male mice can cause transdifferentiation of testicular Sertoli cells into ovarian granulosa cells [25, 26]**.** *NR0B1 (nuclear receptor subfamily 0, group B, member 1*; also known as *DAX1*) is another important factor for ovarian development in the embryo being downregulated in the developing tests. This is supported by duplications at the Xp21.2 locus being associated with 46,XY gonadal dysgenesis. Recently, disruption of the topological-associated domain (TAD) boundary close to NR0B1 has been suggested to be the underlying molecular mechanism [27]. Future understanding of the genetic “switches” of gonadal dysgenesis will significantly profit from a functional association of gene expression with specific cell lineage information at different time points of gonadal development by single cell transcriptomics. Of particular interest will be the in-depth analysis of primary human samples of DSD individuals, such as gonads after surgical removal or tissue biopsies. 

## Hormonal switches of fetal sex development

**Figure 1:**
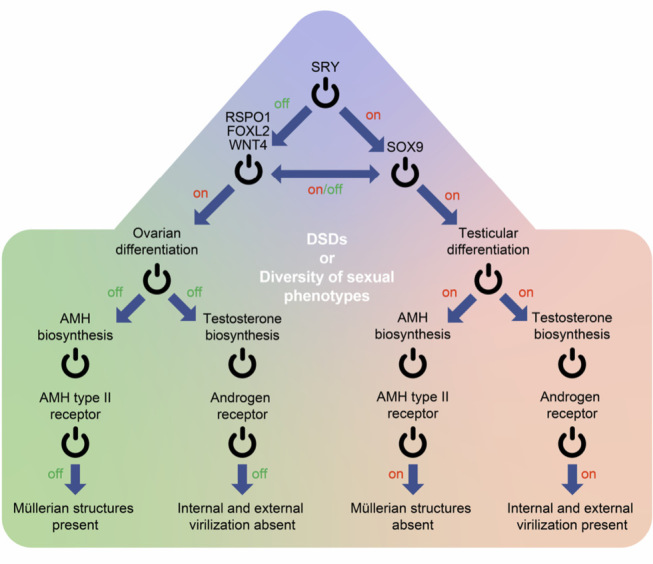
Display of sex determination (gene-controlled development of the gonads) and sex differentiation (hormone-controlled development of the internal and external genitalia) in the form of a simplified “switch-on – switch-off” model of developmental pathways. In case of mutations, many of these “switches” may act variably – virtually more like “sliders” – causing a functional continuum of alterations of gene functions, gene dosages, enzymatic activities, sex hormone levels, and sex hormone sensitivity, giving rise to a broad phenotypical spectrum of biological differences of sex development (DSD) which is a potential model for diversity of genital and somatic sex phenotypes.

During the last century it became evident that sex hormones like the determining genes during gonadal development apparently seem to act like dual “on”/“off” switches for male versus female internal and external genital differentiation (Fig. 1) and that this process occurs between the 7^th^ and 12^th^ week of gestation in the human embryo [28]. High levels of testosterone and AMH produced by the fetal testes [28, 29] lead to male internal and external genital differentiation. The absence of these two hormones typically causes female internal and external genitalia during fetal life. However, in addition to just switching pathways “on” or “off”, rare alterations of sex hormone levels due to different types of gonadal dysgenesis, different specific enzymatic defects or alterations at the sex hormone receptor – and post-receptor levels can lead to a broad clinical continuum of sex phenotypes in DSD. 

## Anti-Müllerian Hormone (AMH) and the AMH type II receptor 

AMH is produced by the Sertoli cells in the fetal testes. It transduces its actions through the *Anti-Müllerian Hormone type II receptor* [30, 31]. In the male fetus, AMH leads to regression of the Müllerian ducts preventing their further development into the upper part of the vagina, the uterus, and the fallopian tubes [32]**.** The absence of high fetal Anti-Müllerian hormone concentrations in the female fetus virtually “switches on” the differentiation pathway of the Müllerian ducts resulting in female internal genitalia (Fig. 1) [32]. The existence of the Persistent Müllerian Duct Syndrome (PMDS) in otherwise normally virilized 46,XY male fetuses underlines the importance of the AMH/AMH type II receptor system as a crucial switch of internal genital development independent of the sex chromosomes. PMDS belongs to the DSD-spectrum and is either due to inactivating mutations in the AMH-gene itself or in the AMH type II receptor gene [33]. Variations of gonadal development due to 46,XY gonadal dysgenesis are another well-known example that supports the role of the AMH/AMH type II receptor system as a developmental switch for internal genital anatomy [34]. Phenotypically female patients with complete 46,XY gonadal dysgenesis show very low AMH plasma levels [35] due to impaired Sertoli cell development in the streak gonads. Therefore, despite 46,XY chromosomes, Müllerian ducts may be partly or completely present in affected individuals. In contrast, in 46,XY DSDs with a high degree of testicularization of the gonads and with significant development of the Sertoli cells, e. g., in androgen insensitivity syndrome, normal-for-male fetal AMH concentrations are usually present. In consequence, Müllerian ducts have typically not developed [34, 36]. In summary, the AMH/AMH type II receptor system is an important hormonal switch for male versus female fetal development of the internal genitalia. Its variations contribute to the continuum of internal sex phenotypes seen in DSDs. 

## Androgens and the Androgen Receptor

Historically, testosterone is the “classical male” androgen and an important hormonal switch of fetal internal and external genitalia development (Fig. 1). If testosterone is synthesized at very high levels between the 8^th^ and 12^th^ week of gestation, i. e., is “switched on” during typical 46,XY male development, a complex program of anatomical virilization of the internal and external genitalia is initiated [28, 37, 38]. On the one hand for the internal genitalia, this involves stabilisation of the Wolffian duct and its development into the epididymis, ductus deferens, Glandulae vesiculares as well as the pars prostatica and pars membranosa of the urethra. On the other hand, for the external genitalia, the genital tubercle and the urethral folds will form the glans penis and the corpus spongiosum. Fusion of the genital midline of the labioscrotal swellings forms the corpus cavernosum and the scrotum. The genital virilization program in males is typically completed in the 12^th^ week of gestation. The absence of very high testosterone levels in the embryo, as in typical 46,XX female development is the sufficient switch to initiate the no less complex anatomically female genital differentiation compared to male genital differentiation (Fig. 1). On the one hand, for the internal genitalia, this leads to regression of the Wolffian ducts. On the other hand, for the external genitalia, the urethral folds will form the labiae minorae. The labioscrotal swellings will become the labiae majorae. In addition, the genital tubercle will develop into the clitoris [28]. 

While testosterone is perceived as the “typical male” sex hormone, estradiol synthesized mainly by the ovaries, is rather perceived as the “typical female” sex hormone, but this is likely an unjustified simplification [39]. It is not clear to which extent estradiol may act as a potential switch for aspects of fetal sex development. In this respect, estrogen receptor expression has been documented in the fetal urogenital sinus, the vagina [40], and the uterus of female human fetuses [41]. Nevertheless, nowadays, fetal ovarian biosynthesis of estradiol is not considered to be biologically relevant. In contrast, it plays crucial roles during puberty in establishing the typical female secondary sex characteristics, i. e., breast development, growth of uterus and Fallopian tubes, as well as female body proportions. More research is needed to understand the potential roles of estrogen for sex differentiation of cells and tissues during fetal life.

The “testosterone switch” is integrated in a network of further upstream – and downstream switches. Testosterone synthesis in the human male fetal testes takes place biochemically in the Leydig cells and is mainly dependent on hCG/LH signaling via the membrane-bound LHCGR (Luteinizing hormone receptor/human chorionic gonadotrophin receptor). While hCG is the most important gonadotrophin in early pregnancy [42], fetal pituitary LH takes over to “switch on” testicular testosterone biosynthesis later [43]. Some years ago, Del Valle and co-workers [44] have published a genomic atlas of human adrenal and gonadal development. They found that LHCGR mRNA is already present in the 8^th^ week of gestation in the human fetal testis supporting the role of the hCG-LHCGR system as an upstream switch for fetal testosterone biosynthesis. They also documented the mRNA of all early steroid biosynthesis enzymes needed for testosterone production, i. e., *StAR (steroidogenic acute regulating protein), CYP11A1 (P450 side chain cleavage), HSD3B2 (3ß hydroxysteroid dehydrogenase, type II), CYP17A1 (17 alpha hydroxylase/17,20 lyase) and HSD17B3 (17ß hydroxysteroid dehydrogenase type III)* [44]. Interestingly, recent single cell RNA sequencing data reveals that, although all steroidogenic enzymes necessary for androgen synthesis are present in the fetal testis, there is a compartmentalization of the final step of testosterone conversion by *HSD17B3* to fetal/immature Sertoli cells [45]. This compartmentalization has been observed before in mice [46], but was not expected in humans. In the adult, *HSD17B3* expression switches into Leydig cells, but converting androstenedione to testosterone might be mainly based on its isoenzyme *AKR1C3 (HSD17B5)* at this time [45]. The reason for this compartmentalization, which is a challenge to the classical role of Sertoli- and Leydig cells in testicular steroid biosynthesis, is not clear to date. Still, it may have enormous relevance in explaining clinical and biochemical observations in certain DSDs, like the significant pubertal virilization in HSD17B3 deficiency. 

The importance of the combined hCG-testosterone system as a crucial combined switch for sex differentiation is underlined by DSD individuals with inactivating molecular variations in several of the involved genes, e. g., in Leydig cell hypoplasia [47], *StAR* deficiency [48], *P450scc* deficiency [49], *3ß-hydroxysteroid dehydrogenase type II* deficiency [50], *17alpha/17,20 lyase* deficiency [51], *17ß hydroxysteroid dehydrogenase type III* deficiency [52] and *5alpha-reductase type 2* deficiency due to mutations in the *SRD5A2* gene [53, 54]. The latter DSD is especially important since it demonstrates that the most active androgen dihydrotestosterone (DHT), but not testosterone alone, is the biochemical master switch needed for regular external genital virilization in males.

During recent years, the story of androgen biochemistry became more complex due to the (re-) discovery of seemingly “forgotten” androgenic steroids [55] and due to the discovery of previously unrecognized biochemical pathways to DHT, e. g., the **“**backdoor pathway” [56]. The “backdoor pathway” starts with 17-hydroxyprogesterone being reduced by 5α-reductase and 3αHSD to 5α17-hydroxypregnanolone. The latter, in turn, undergoes 17,20-lyase reaction to androsterone. Reductive 17βHSD activities and oxidative 3αHSD activities then lead to dihydrotestosterone. Swart et al. [55] have described a pathway generating “11-oxygenated C19 androgens”. This pathway starts with 11β-hydroxylation of androstenedione and testosterone to their respective 11-oxygenated-forms. They are activated in peripheral tissue to their 11-keto forms by *HSD11B2* [55]. The role of these and other alternative androgen pathways as modifiers or even switches in male and female fetal sex development is sparsely understood. Flück et al. [57] described inactivating mutations in the *AKR1C2* gene in individuals with partial lack of genital virilisation**,** suggesting a role of the “backdoor pathway” in DSDs and potentially sex diversity of phenotypes. In 46,XX DSDs like 21-hydroxylase deficiency, 11-oxygenated C19 androgens have been suggested to contribute to the sex phenotype [58]**.** More rigorous biochemical research on genital tissues and cells in control individuals and defined DSDs is needed to understand the complex architecture of the “androgen switch(es)” in DSDs and their potential role(s) in diversity of sex phenotypes. 

**Figure 2:**
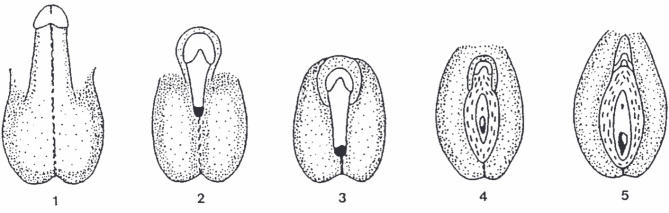
Classification of phenotypes in androgen insensitivity syndrome ranging from minimal androgen insensitivity syndrome (MAIS) with normal external virilization (1) over different types of partial androgen insensitivity syndrome (PAIS) (2–4) to complete androgen insensitivity syndrome (CAIS) (5) with completely female external genitalia [61]. The figure also illustrates the continuum of external virilization in AIS due to a continuum of functional variability at the level of the androgen receptor in addition to a simplified “on-off-model”. Reproduced with permission from Springer Nature.

The intracellular androgen receptor (AR), which is a ligand-activated transcription factor of androgen-regulated target genes is the key molecular switch for virilization of the fetal genital target tissues [36, 59]**.** Binding of testosterone or dihydrotestosterone induces specific structural changes to the AR, recruitment of co-regulators, translocation into the nucleus, binding to specific androgen-response DNA-elements, and finally lead to transcription and translation of androgen responsive target genes [36, 59]. The critical role of the AR as a molecular switch is confirmed by 46,XY DSD individuals who have inactivating mutations of the X-chromosomal *AR-*gene leading to the androgen insensitivity syndrome (AIS) [60]. This ranges from complete androgen insensitivity (CAIS) syndrome with female external genitalia to partial androgen insensitivity syndrome (PAIS) with different variations of hypospadias to individuals with only minimally reduced virilization (MAIS) (Fig. 2) [36]. 

Interestingly, more than 30 years since molecular cloning of the AR-gene [60], there is still only very sparse knowledge on the androgen-dependent gene transcription program in human sex differentiation, which is in contrast to genome-wide transcription data in prostate cancer [62]. In rodents, a few androgen-regulated morphogens have been published (*FGFR2, FGF10, MAFB, smooth muscle actin*) [63–65]. Amato and Yao [66] used single-cell RNA sequencing during sex differentiation to identify the main cell populations and their lineages in the external genitalia in wild-type mice at embryonic days E14.5, E16.5, and E18.5. The authors identified the general cell types in the external genitalia of both XY-males and XX-females. However, due to the XX-female versus XY-male experimental scenario excluding DSDs, e. g., XY-female developments, the downstream programs switched on via androgen and the AR could only indirectly be concluded. In 2009 we discovered *Apolipoprotein D (APOD)* to be strongly androgen regulated in human male cultured genital skin fibroblasts, which is not the case in 46,XY-females with CAIS due to proven inactivating mutations in the AR-gene [67]. We currently have no data supporting that *APOD* itself may be a downstream switch for genital differentiation (unpublished). However, using APOD as a molecular marker in an in-vitro test developed in our laboratory in Kiel (“APOD-test”), we could identify a subgroup of typical clinical AIS-patients having no inactivating mutations in the coding regions of AR-gene (AIS type II) [68]. AIS type II as a specific newly defined group of DSDs, somewhat opened a scientific door to enhance understanding of the role of AR as a molecular modifier and switch of sex differentiation and diversity of sex phenotypes in humans. At the level of the AR-gene itself, we could identify a previously unknown AR-promoter region in which specific patterns of increased methylation caused decreased AR mRNA transcription and were associated with clinical AIS type II [69]. Very recently, we showed that the formin and actin nucleator *DAAM2 (Dishevelled Associated Activator of Morphogenesis 2)* is necessary for androgen-regulated nuclear actin assembly and hence, for regular AR mediated gene transcription [70]. This underscores the molecular complexity of the function of the AR as a “switch” for sex differentiation. Since about 60–70 % of patients with partial AIS type II currently have no proven mutation in the AR gene [36], there is a considerable likelihood for the existence and future identification of more genes involved in regular function downstream of the AR in human sex differentiation. 

## Trans-omics aspects for understanding the genetic and hormonal switches of fetal sex development

The concept of molecular switches, including genetic or hormonal switches, has been extensively studied before. Simple signaling pathways can be embedded in networks that generate complex – multistable behavior through non-linear positive and negative feedback regulation. In analogy to electric circuits, this can generate oscillators, toggle switches, and hysteresis behavior [71]. Examples thereof include chemotaxis, cell-cycle checkpoints, and in particular, development [72–74]. Models of molecular interaction can explain how dysfunction in proteins or interactions lead to switching a system from mono- to bi- and even multistability, and how mutations in regulatory pathways lead a phenotypic variability in the stable configurations of such a dynamic system. Multistability refers to the system property of having more than one stable state. E.g., in a system of mutually repressing genes, this can lead to either one being expressed while the other being repressed. An unstable equilibrium must exist between the stable states, disallowing the two genes to be simultaneously expressed. Mathematically, protein networks can be deduced using the law of mass action for protein networks, which allows direct causal deduction of principles. However, such networks are hard to scale up to the level of whole cells or even organs. To simplify chemical details, logic or Boolean models have been introduced that capture the, mostly binary logic of abstract molecular interactions [75]. Such logic models can provide insights into the molecular causes of gonadal or ovarian tissue development. Rios et al. [76] have developed a Boolean network describing the gonadal sex determination based on Sertoli progenitor and granulosa progenitor cell fate decisions. Using model simulations, they could not only define necessary regulatory interactions to activate the sex-determining region Y or the WNT4/ß-catenin pathway, leading to either testes or ovary. They also found a pattern of altered interactions resembling a disordered ground reminiscent of DSD. 

Apart from detailed dynamic modeling, complex biological processes have been explored on the various Omics levels in biology, from the genome, transcriptome and epigenome, to the proteome, metabolome and microbiome. The idea is to unravel the interrelationships of the involved molecules and their pathways [77, 78]. Data integration tools for high-throughput data combined dimensionality reduction, multi-variate regression tools, and network and Bayesian approaches [79]. Multi-Omics analysis has spearheaded the field of oncology with tools for pan-cancer studies and data integration, such as the Cancer Genome Atlas or cBioPortal [80]. Integration of genetic, epigenetic, and prior pathway information has led to the discovery of novel biomarkers and patient stratification and explained cancer origins [81]. Yet, few multi-Omics studies on gonadal development in humans DSD exist, but first results are promising, particularly in single-cell analysis and spatial transcriptomics, that capture the biological variability, cell types, and spatial interactions all of which determine the phenotype in gonadal development. Garcia-Alonso et al. published a comprehensive single-cell and also spatial transcriptomics map. Comparing the data in humans to that in mice, the authors defined germline and somatic cell states and spatial expression of individual transcription factors and their programs that control sex-determining factors [82], showing how novel regulatory schemes can be discovered through insights into the spatiotemporal organization of gonadal tissue and individual cells. 

Thus, the idea of genetic and hormonal switches leading to non-binarity of sexes is long known in the Systems Biology community. However, its proof and applicability to the bedside have been lacking due to the many ethical, technological, and clinical obstacles. With the novel technologies in sequencing and high-throughput cellular resolution at the molecular and spatial levels, the idea of genetic switches in sex development should and will be certainly revisited. This will not only enhance in-depth understanding of DSDs but in addition many diagnostic and therapeutic processes in medicine by an improved broad consideration of sexual diversity and the underlying mechanisms in the future. 
